# “We forgot about the donkeys!” An institutional analysis of the shift in animal welfare from direct implementation towards advocacy-based programming

**DOI:** 10.1017/awf.2024.11

**Published:** 2024-02-14

**Authors:** Emily Haddy, Leanne Proops, Faith Burden, Zoe Raw, Juliane Kaminski, Julia Brown

**Affiliations:** 1 University of Portsmouth, Faculty of Humanities and Social Sciences, Portsmouth, Hampshire, UK; 2 University of Portsmouth, Department of Psychology, Portsmouth, Hampshire, UK; 3 The Donkey Sanctuary, Equine Operations, Sidmouth, Devon, UK; 4 University of Portsmouth, School of the Environment, Geography and Geosciences, Portsmouth, UK

**Keywords:** Animal welfare, institutional analysis, NGO, organisational change, welfare initiative, working equid

## Abstract

Shifts from direct implementation to advocacy-based programming have been documented across many non-governmental organisation (NGO) sectors, including animal welfare. Semi-structured interviews with 32 staff from different positions within animal welfare NGOs explored recent programming changes. Maintaining a balance between direct implementation and advocacy-based activities emerged as a strong theme. The findings suggest that risks are associated with both the direct implementation *status quo* and transitioning to an advocacy-based focus. Risks of the former include treating symptoms rather than root causes of welfare problems. Organisational change can be disruptive and necessitates realignment of core competences, in turn influencing NGO mission. Identified risks of transition include loss of individuals whose values fail to align with new programming directions, increased upwards accountability requirements for accessing institutional donors and difficulties when phasing out direct implementation approaches. Whilst having to be dynamic, NGOs need to evaluate the risks associated with programming decisions, considering their vision, mission and staff identity in order to ensure that welfare programming is as effective as possible.

## Introduction

Any NGO that scales up their operations or decides to pursue a change of programming direction, often in the form of a shift from traditional, direct implementation to more policy-advocacy work, will undergo some form of organisational change and governance restructuring (Billis & MacKeith 1992). This process may take the form of a change of emphasis over who the intended beneficiaries of the interventions are. In the case of working equid programming, this results in a move away from veterinary clinics towards a focus on owner livelihoods and more holistic programming and advocacy work. As the organisation changes it goes through a process of disruption and transformation (Kuruppu & Lodhia 2019) and as the core competences of the organisation evolve (the knowledge, skills and abilities that organisations are known for) so too does the character of the organisation, which in turn influences the values of the mission of the NGO and ultimately its sense of purpose (Berny 2018). While this shift away from an on-the-ground welfare interventionist focus within the working equid sector may address concerns over the sustainability of programming at some levels within an organisation, for those with a clear animal welfare motivation, this may represent mission drift and an undermining of the original focus of the charity which was to alleviate equid suffering. As one respondent suggested in our study, in the process of transformation “*[w]e forgot about the donkeys!*” This may also erode the individual donations upon which the sector has been heavily reliant.

Undertaking an institutional analysis and going down the organisational hierarchy of eight animal welfare NGOs with working equid programmes, from both high-income countries (HICs) and low-middle-income countries (LMICs), affords us the opportunity to explore the varying views and attitudes of officials at different levels within the organisations. These examine the changes in core competences and resultant impact on the character of the NGO that have occurred, or that some organisations are on the cusp of, and what these may mean for the trajectory of the organisation and sector. It also allows us to explore the internal implications of policy change: are all levels within the NGO aligned with the change in direction? Are there issues, constraints and potential risks by not reforming, as well as risks associated with transformations away from a purely direct implementation focus? Do governance structures facilitate the transition or are they out of kilter with the new policy direction? Further, by considering perspectives from all levels within an organisation, we hope to be able to contribute to what the optimum balance of programming emphasis may be on the spectrum of equid welfare direct implementors through to advocacy-only.

The changes that animal welfare NGOs are on the cusp of or have gone through, mirror the earlier changes within development (Hudson 2002) and environmental (Berny 2018; Berny & Rootes 2018; Levine 2002) NGOs in the 1990s and early 2000s, of a shift away from direct implementation to more advocacy work, and within environmental NGOs more specifically a shift from single species to more holistic programming (Levine 2002). It is widely accepted within the sector that working equid NGOs are lagging behind the broader change that have seen NGOs cede to the pressure to adopt advocacy over direct implementation, thus this study is able to draw interesting parallels with and lessons from earlier transformations in allied NGO sectors.

The strength and value of this paper is that these kinds of insights, following an in-depth study of internal operations and governance structures of the NGO sector, remain relatively rare within the academic literature (Billis & MacKeith 1992; Kuruppu & Lodhia 2019). Lewis and Opoku-Mensah (2006) argue that NGOs should be seen as subjects for research because of the important role they play in processes of change. Change within NGOs is still an under-researched topic, despite being the recipient of a fifth of global funding from governments (Kuruppu & Lodhia 2019); partly because it can be difficult to gain access as NGOs, in a competitive funding climate, may be reluctant to air internal discussions. The contribution of this paper is that authors LP and EH have a long-standing collaborative relationship with the NGOs, including international non-governmental organisations (INGOs) and partner organisations; second, the working equid sector is undergoing some significant changes in policy, and is keen to learn from other NGO sectors who have undergone change earlier on; third, a key motivation is to learn from the experiences and perspectives of officials with a variety of roles and responsibilities at different levels as well as length of time with the organisation.

Drawing on original data, comprising 32 in-depth, semi-structured interviews with representatives at different levels within eight animal welfare NGOs, subject to rigorously applied thematic analysis, this paper seeks to explore what happens when organisations embark on a change of emphasis and to understand how people working at different levels within organisations feel about these changes. Through the lens of organisational change, we consider both the risks as well as potential benefits. The paper proceeds with a review of the changing roles and focus of NGOs with particular focus on the lessons to be learnt from earlier transformations, of the impact on the competences and character of NGOs, before focusing on working equid programming. We then set out the data collection upon which the institutional analysis of the sector is based, and share the results organised around the risks of not evolving followed by the identified and potential risks of accepting change.

## The evolving NGO landscape and the impact on competences and character

### Changing NGOs

The NGO sector has grown, in terms of membership, funding and scope, since the 1970s. This often mirrors political changes in both HICs, with the adoption of neoliberal agendas, and LMICs, as a legacy of the Washington Consensus. NGOs have thus sought to fill service gaps left by the cessation of state services and have seen their funding increase as both their scope of intervention and field of influence increased (Berny & Rootes 2018). In parallel there have been calls to professionalise their activities, in part because as their funding base has broadened to include institutional donors and governments, they needed to demonstrate upward (to donors) accountability of received public funds. Increasing funding has seen an increase in the level of scrutiny, and the need for more rigorous monitoring and evaluation systems and reporting procedures (Tandon 2000; Wright 2012). The 1990s thus became the era of consolidation and institutionalisation: many environmental NGOs with radical beginnings withdrew from direct action, formalised their structures and became mainstreamed (Berny & Rootes 2018; Hadden & Bush 2021).

One route to securing funding in the 1990s was for NGOs from different sectors to collaborate, for example, conservation and development NGOs. The forging of strategic alliances for more integrated community-based and livelihood programming has been subject to debate, with concerns raised that conservation NGOs were diluting their conservation mission and legitimising funders such as the World Bank at the expense of the NGOs’ reputation (Levine 2002). The donor-recipient relationship can also undermine the perceived neutrality of the NGO in advocacy work, hence undermining the effectiveness of its efforts (Levine 2002). However, there are significant benefits for collaboration, notably accessing new sources of funding and a livelihoods focus seeing greater local ownership and control and an increase in incomes which are viewed as central to more sustainable programming (Levine 2002). Pacheco-Vega and Murdie’s (2021) research indicates that the success of advocacy work by environmental NGOs and their ability to influence is largely dependent upon the inclusion of local participants in advocacy work and the extent to which the state is susceptible to international pressure.

From Hudson’s (2002) work on transformations within HIC-based development NGOs in the 1990s, important lessons emerged around the themes of differentiation and collaboration, legitimacy (of the shift towards advocacy), representation and accountability. Research with development and environmental NGOs indicates feelings of betrayal as NGOs made the shift away from direct action (and in the field of development the shift from direct implementation and service delivery) towards lobbying (Berny 2018). As key skills and approaches have evolved, with increasing institutionalisation, so have the types of job roles recruited for and thus the character of organisations and their mission. This process radically changed the ethos of many from being viewed as radical, direct-action groups (potentially illegal activities) to legal “sell outs” (Berny 2018). Directors of NGOs have argued that in protecting the long-term financial sustainability of the organisations they have been prepared to make compromises that others within their own organisations have been less ready to accept (Berny 2018). It is widely accepted that to secure future institutional funding, NGOs place a lot of emphasis on keeping their donors happy (Martin & Brown 2021) or seek to forge strategic alliances (Levine 2002).

Understanding the dynamics between HIC based NGOs and their LMIC partners is another important dimension: a change in emphasis and governance at the former will impact on the latter (Hudson 2002; Mitlin *et al.* 2007; Hadden & Bush 2021). As Mohan (2002) suggests, this relationship is complex because the latter lend ‘credibility’ to international operations, and HIC-based NGOs are particularly beholden to the agendas of their donors and the need to impose monitoring and evaluation procedures, and thus often push these onto local partners (and their own local field staff); power relations are rarely balanced.

We argue that important lessons from earlier experiences of this evolution from service delivery to a hybrid or advocacy focus can be considered in our examination of the current transformations within the animal welfare sector and the impacts on individuals within these organisations.

### Animal welfare NGOs: A spotlight on working equid programming

Globally, working equids (donkeys, mules and horses) provide essential support to an estimated 600 million people (Sommerville *et al.* 2018). They can provide a source of income, a form of transport and access to basic necessities such as water and firewood (Pritchard 2010). Over 85% of the world’s equids are found in low and middle income countries (LMICs) where they are owned by some of the poorest members of society (Burn *et al.* 2010; Stringer 2014). Despite their value, socioeconomic limitations mean that owners are often unable to provide adequate nutrition, veterinary care and working equipment for their animals. Research from a range of countries has shown that welfare problems, such as lameness, wounds, poor body condition and dehydration are common (Burn *et al.* 2010; Pritchard *et al.* 2005, 2008; Tesfaye & Curran 2005; Reix *et al.* 2014).

Within the animal welfare NGO sector, working equid programmes are relatively niche in focus. They are known for their key competency in promoting positive welfare and providing veterinary clinics in countries with low equid welfare standards. Traditionally they are direct implementors: the target of their mission was to alleviate suffering of working equids – either working directly or via partner organisations. This welfare focus was clearly relayed to their donors (often via the effective use of emotive adverts): the sector was traditionally self-contained in terms of its funding base which consisted primarily of individual donors; therefore the NGOs running working equid programmes had not needed to reach out to institutional donors (Upjohn *et al.* 2014). The working equid sector had a clear sense of purpose and alignment of their mission with their programming. It is an opportune moment to investigate the working equid sector because internal evaluations of programming have raised questions over the long-term sustainability, effectiveness and reach of direct implementation welfare interventions (Haddy *et al.* 2022).

The One Welfare approach (García Pinillos 2018) has been developed in recognition of the benefits of a more holistic approach to considering problems involving animal welfare, human well-being and the environment. In terms of equid welfare programming, this involves working with equid owners and emphasises the link between improved equid welfare and more productive livelihoods as a route to realise better welfare standards. However, a shift away from veterinary clinics to integrated approaches encompassing the central tenets of the One Welfare approach would see some NGOs as no longer direct welfare implementors – they may now partner with a development NGO in a broader consortium. Whilst other NGOs may evolve their remit with a change of emphasis to programming that focuses on livelihoods and community development and the adoption of participatory approaches, it is very important to recognise this may not be the background of some field staff and requires a mindset change and a different skill set (for example, in community development, community engagement and training). This may result in staffing changes bringing in competencies other than animal welfare, for example in social sciences and economics, both for on-the-ground programming and middle and senior management level, in a bid to adopt best practice and avoid a mismatch of required skills.

Informed by discussions with representatives of the NGOs included within this study, as well as extensive professional involvement of several of the authors within the equid sector, we argue that the shift from direct implementation approaches to more holistic programming represents a significant evolution of core business and wholesale mission change as the core competences of the organisation evolves along with its character. In some instances, this has also required the NGO to broaden its funding base away from reliance on individual donors and secure institutional donor backing. This period of introspection and reflection has led some NGOs to conclude that their efforts would be more effective by becoming advocacy-only organisations and completely withdraw from direct implementation or to prioritise national lobbying to raise the agenda of equid welfare to effect policy change. This also mirrors identified changes within environmental NGOs more broadly (Berny & Rootes 2018). Thus, similarly, whether animal welfare NGOs form collaborations with bigger development NGOs, broaden their remit and thus need to appeal to institutional donors, or reconfigure their mission to be advocacy-only which would also appeal to institutional donors, all these approaches would result in needing to professionalise operations and develop stronger governance and accountability structures and mechanisms. As will be evidenced in the *Results and Discussion*, the transitions outlined above evoke strong feelings amongst NGO officials: staff are passionate about animal welfare and for many this was their motivation for joining the NGO. The sector is thus on the cusp of major disruption and transformation with a refocusing of activities and the inevitable changes to operations and governance structures.

## Materials and methods

The University of Portsmouth’s Ethics Committee for the Faculty of Science and Health reviewed and approved the study (reference SHFEC 2020 – 087) and informed consent was obtained from all participants prior to being interviewed. A total of 32 in-depth semi-structured online interviews were undertaken between February and July 2021 with officials representing eight animal welfare organisations with working equid programmes across 13 countries. These comprise four organisations that would be classed as INGOs (and are at different stages of transition); three local partner NGOs and one organisation that works across the sector. Participants from a range of positions within NGOs were sought, including Directors of Research and Operations, Managers of Regional Operations, and Researchers and Welfare Officers working on the ground in equid owning communities. This range of roles was deemed representative of the welfare initiative process, from the direction and design of initiatives to their physical implementation in equid-owning communities. Further, organisational change also has varying implications for staff at different levels which is something we were keen to explore.

Interviews were conducted and recorded via Zoom video conferencing software, lasting an average of 55 min (range 30–108 min). Interviews were conducted in English, a language in which all participants were fluent (with the exception of one interview which was conducted in Spanish, the participant’s first language, with a translator present who translated to English in real-time). The purpose of the subsequently anonymised interviews was to garner participant views and personal experiences regarding: the design and implementation of different equid welfare initiatives, factors affecting initiative success (or failure) and the changes experienced or planned for in organisational programming and mission over time (for full details, see the interview guide in the Supplementary material). The interview recordings were transcribed, first via an automated transcription programme Otter.ai (Otter.ai Inc 2020). Transcripts were subsequently checked by EH against each recording and edited to correct for any transcription errors, this process also served as the familiarisation stage of analysis. The interview data were subjected to thematic analysis, undertaken according to the phases of Braun and Clarke (2006) using a semantic approach to identify the common themes occurring across both different organisations and across different job roles. EH reviewed all transcripts and generated initial codes manually using coloured pens to highlight different subjects discussed and within them common concepts that appeared repeatedly across manuscripts. Based on these codes, a list of potential themes were compiled in MS Word (Microsoft® 2023). These potential themes were examined by two other authors (JB and LP) who reviewed (and merged or separated) themes until a consensus was reached. EH then compiled representative quotes from participants that illustrated the various points made within each theme and presented them within the narrative. Themes (and subthemes) are presented as headings in the *Results and Discussion.*

## Results and Discussion

The NGOs in our study are at varying stages of evolving away from direct implementation initiatives towards more holistic livelihoods-based programming and incorporation of more advocacy work or are moving towards being completely advocacy focused. The two main themes: the risks of direct implementation approaches and the development of more diverse programming, reflected this shift with four subthemes identified under the second theme (the development of more diverse programming): more community-focused programming, increased partnership working and accountability, impacts on staff of programming changes and changes to organisational structure and programming autonomy. This section outlines and discusses these thematic areas.

The breakdown of participants was as follows: senior management (SM): eight (from four INGOs), middle management (MM): 13 (from seven I/NGOs), field staff on the ground (FS): eight from four INGOs, other (atypical roles) (O): three from one INGO. NB the only identifier used are these categories, not the organisation that the participant works for.

### Risks of direct implementation approaches

Equid welfare programming traditionally aims to support owners and improve standards of working equid welfare. A variety of approaches have been utilised over time in order to try and achieve welfare improvement, but concerns have been raised about the long-term efficacy of the approaches implemented. Service provision approaches (those that give a service, often for free), such as farriery services, feed provision or free veterinary clinic models whereby owners can access treatments ranging from preventative to emergency care, were previously the most common (Upjohn *et al.* 2014). However, these approaches have been criticised for a number of reasons. The offering of a free service has the potential to create a dependency upon this service which may later be withdrawn (Upjohn *et al.* 2014). Participants described the need to ensure that other measures were in place if considering a free clinic approach to prevent this situation: *“I think it depends what you are doing that alongside to ensure that people don’t develop a dependency on that free service”* [P2 FS]. There has also been concern expressed that service provision approaches were treating the symptoms of welfare issues rather than preventing their root causes (Rogers 2010).

There was widespread agreement that accessing treatment could significantly improve the welfare state of the animals treated and acknowledgement that creating such an immediate change was fulfilling for staff: *“[t]here is part of me that loves doing that…because for us I think that feels great – we have fixed an animal that’s fantastic”* [P2 FS]. However, there was also acknowledgement that the ‘fix’ was likely to be short-term and that the access to free treatment may impact on owner motivation to make long-term welfare-positive behavioural changes: *“[i]f you’ve got something that can mend your animals you might not be so keen to prevent that issue in the first place”* [P5 MM]. In order to make lasting changes to owner management behaviours, programmes need to foster more than a temporary engagement with equid-owning communities (Pritchard 2010). A middle management interviewee (P21 MM), reflecting on the traditional mission of the organisation and their adoption of a free veterinary clinic approach summarised that: *“They would… love to work just with animals because animals are easier. But, but no, we have to work… with humans and all that entails”.*

When discussing service provision, it was commented that *“[y]ou’re not actually setting up the country to look after itself. So it’s not really future proofing. And it’s probably not best use of your money because you can only…improve the lives for a limited number of animals”* [P14 SM]. With long-term sustainability and scale in mind, participants were increasingly questioning the potential impact of programming options: *“[h]ow is this creating a lasting change for generations of donkeys to come rather than just the one that you see today?”* [P10 O]. Investing instead in supporting existing in-country infrastructure such as veterinary capacity, farriery and saddlery services were described as a more sustainable option, this also mitigated another associated issue – that of free services undermining existing local service providers: *“[y]ou are also doing a lot of unintended impact in the veterinary community. Local veterinarians that are working in the country, they also provide the services. So you have to be very careful”* [P21 MM].

The identified risks of not changing the focus of activities away from purely service provision approaches have been outlined, in the following section we explore the benefits of reformulating programming focus, as well as the perceived risks of not retaining some direct welfare implementation.

### A new focus: The development of more diverse programming

In response to these concerns and evaluations of previously run initiatives, NGOs have been revising aspects of their equid welfare programming and approaches (Mohite *et al.* 2019). Subsequently, as a sector, animal welfare NGOs with working equid programmes are undergoing significant organisation and mission change. With the creation of lasting change, a driving force behind programmatic approaches, organisations are transitioning to more interdisciplinary programming which seeks to actively engage with the people who influence the welfare of working equids, whether they be equid owners in the community, local veterinary students or politicians developing agricultural and animal welfare policy.

This shift reflects movement within organisations from a single animal welfare focus to a more holistic perspective, taking into account the relationships between animal welfare and other sectors. For many organisations this can also involve a transition from reliance on individual donors to seeking of larger institutional donors in order to realise broader, more interdisciplinary programming.

### More community focused programming

At the community level, equid welfare programmes have, over time, adopted a wider range of approaches, particularly adopting methods that place an emphasis on community engagement. Approaches such as community participatory exercises (activities that community members work together to complete, using their local knowledge and perspectives) were felt by participants to be effective in engaging individuals, from the initial process of identifying priority welfare issues within the community to evaluating the success of implemented initiatives. A field-based participant suggested:

*“[m]any, many years ago, people or researchers imposed their own opinion on the communities, …I think they don’t actually ask their opinion… they don’t actually give them a chance to be a problem identifier or a problem solver. So, that was a big problem, nowadays that is actually changed… community engagements is very, very important”* [P11 FS].

Participants also discussed that ideas generated by the community themselves were more likely to be successfully followed though and enabled communities to feel a sense of ownership of, and responsibility for, change which did not exist with previous service provision approaches. A field-based participant summarised this view: *“[t]he fact that they* [the community] *came up with it and they were leading it meant that I had way more faith that it was going to work!”* [P2 FS]

It was apparent from the interviews that there were considerable benefits associated with more collaboration and holistic programming. Reflecting the new focus on the human influences on animal welfare and an increased level of interdisciplinary working, three organisations discussed early-stage initiatives that focus exclusively on the owner. These aim to diversify livelihoods within equid-owning communities, so they are less dependent on their equids. Increased livelihood diversification has also been linked with increased income levels and greater community resilience (Pasteur 2011; Velázquez-Beltrán *et al.* 2011; Ainuddin & Routray 2012). There is hope that these initiatives will therefore indirectly increase equid welfare within target communities: *“[i]f there’s an extra source of income…there’s less demand placed on the horse. And if we need to rest the horse we will rest the horse because we don’t absolutely need to work there. That’s a massive shift for* [us]*”* [P13 SM]. However, one management participant, with a veterinary background, expressed concern that initiatives not directly targeting equine welfare may not be received as well by individual charity donors, who may think *“[o]h, you’re just spending money on strengthening their livelihoods. If you* [i.e. the donor] *wanted to do that, maybe you’d be donating to Oxfam, for example”* [P20 SM].

### Increased partnership working and accountability

Interdisciplinary collaborations were also discussed in reference to the increase in partnerships formed with other types of NGO. Future collaborations between animal welfare NGOs and human development organisations were suggested but were still in their infancy. The versatility of working equids and the range of benefits they can bring to communities mean that equid welfare programmes can find common ground within a wide range of humanitarian agendas. The interconnectedness of equid welfare, human well-being and the environment is clear when considering the role equids can play in working towards UN Sustainable Development Goals (United Nations 2020). For example, equids can play a role in reducing poverty, providing access to clean water, offering a sustainable source of energy and enabling resilience to climate shocks and extreme weather events (The Brooke 2019). A middle management participant summarised the benefits of this approach: *“[w]e can integrate the equine welfare aspect from gender aspect, you can integrate the equine welfare aspect with educational aspect, we can integrate equine welfare aspect with food security aspects”* [P30 MM]. The benefits of potential partnerships with in-country enforcement bodies such as governmental welfare inspectors, police officials or established organisations from the international development sector were described as access to larger amounts of funding for joint projects, a large increase in potential reach and the utilisation of multidisciplinary skill sets, benefits of collaboration also described in the environmental NGO sector (Levine 2002). A manager with international development experience suggested *“*[we are] *quite an immature international organisation so going and working with partners that have that credibility in the space using our resources to create impact for working animals is quite a smart way forwards”* [P7 SM].

One consequence of the described increase in partnership working, especially when collaborating with larger organisations in the development sector, is an increased demand for formal monitoring and evaluation (M&E) processes, in order to create a more structured, strategic approach and aid in budgeting and programming decisions (Upjohn *et al.* 2014). A lack of systematic M&E has been previously highlighted as a problem in the field of equid welfare (Upjohn *et al.* 2014) and interviewees such as P7 expressed the view that the animal welfare sector was roughly a decade behind the humanitarian sector in its evaluative processes. The nature of equid welfare funding being generated mainly through individual rather than institutional donors has meant that up until recently there has been little of the pressure seen in the humanitarian NGO sector to account to donors through evaluation (Upjohn *et al.* 2014). However, as one participant suggested:

*“I think* [our organisation] *is wanting to attract proper funding, bilateral funding from the human development sector and you have to have things in place to be able to do that. Like reporting frameworks, due diligence must be met in terms of requirements, it means you have to have a lot more structure in approach and I think that has been quite tricky for some organisations who have been used to a much freer sort of laissez-faire relationship”* [P15 MM].

Thus, a relatively recent shift occurring across equid welfare organisations has been to systematically improve M&E processes, to meet donor eligibility criteria and for organisations to ensure that initiatives are achieving their intended strategic impact.

Participants acknowledged that creating lasting change through programming takes time, especially in work with communities where effective relationships need to be built. As one middle management participant reminded us:

*“[i]n working with communities, it’s one of the challenging issues, but at the same time, rewarding, depending on how you approach it. As you know, behavioural change is a time-taking one, it doesn’t come as easily as we would like it to happen. Because convincing…communities to change their mind, …practices prevailed for so long, is really a process of convincing someone to change, you know, their belief systems”* [P29 MM].

However, this time investment is often at odds with accountability requirements of the new institutional donors requiring evidence that the initiatives they are funding are achieving results. A middle management [P21] interviewee felt:

*“I think, for us working in the field, we need to be very, very clear with the people from the donor organisations, people that are not from the country…tell them that…this is going to take time. I know that donors are always wanting to have, you know, result, result, result. But when you’re working with human beings, I mean, things take time”.*

Further, engaging with participatory approaches, as another middle-level participant [P6] pointed out, means “*I can’t tell you what I’m going to get because I need to go and sit on the ground and talk with the community, find out where they are”.* Whilst participants appreciated the need for monitoring initiatives, it was felt that trying to meet short-term accountability targets within a long-term process can create pressure for individuals implementing initiatives. *“I think it is very, very common for people to be under pressure to show results”* [P5 MM].

Some NGO officials still felt responsibility to their individual animal welfare donor base who also want to see immediate tangible outputs that are directly addressing equid needs. However, as discussed, NGOs are increasing the number of community-focused programmes with a view to creating sustainable impact. As middle management P21 suggests: *“[y]ou know that long-term, this* [community-based] *is the best action to carry out. But…those people want you to help the animals directly, providing medicines and feed and that’s what brings a lot of* [individual] *donors.”* This tension between the changing competences of the organisation, following reprogramming and professionalisation and accountability procedures, and the resultant character and mission evolution sits uncomfortably with some field staff: *“[i]t’s great that we took accountability to the donors so seriously, but we ended up being so careful about accountability that we forgot about the donkeys”* [P4 FS].

### Impacts on staff of programming changes

New programming directions can mean huge changes in the way that staff on the ground are working. However, moving from reactive service provision to more proactive livelihoods approaches can be difficult to adjust to, especially for field staff, often with a veterinary background, who have previously been able to provide an immediate reduction to animal suffering. *“It’s been quite hard for the team to change their tune as well because they are on the ground and it’s a harder kind of work in a way, because immediately resolving a problem… you’re out there seeing stuff but it’s a feelgood factor quicker. You’re resolving a situation… whereas it’s much more uncomfortable I would say this* [new] *kind of work”* [P9 FS]. Broadening organisations’ focus to increase the amount of work done with people means that as a consequence less time is spent directly improving welfare on the ground through service provision. As was found with studies looking at the earlier transformation that development and environmental NGOs went through (Berny & Rootes 2018), the changes in equid NGO programming divided opinion of some staff who were not aligned with the new direction:*“[o]rganisations are made of people. And so, it is very difficult for an organisation that has, you know, some type of history with the same people… for them to change their minds”* [P21 MM]. Field staff especially were concerned that new approaches may not be as effective, and that mission drift would mean that immediate equid welfare needs would be too sidelined within the new direction.

Unfortunately, it was acknowledged that a change in philosophy often meant a change in personnel, with some long-established individuals who were not able to reconcile with this change in mission either choosing to leave or losing their positions. In restructuring, a senior manager [P27] summarised: *“[s]ome were really pro…and people on the ground had been championing the move* [away from vet clinics] *for a long time, and were really pleased to see it. Others disagreed. And that conflict was managed and some people left”.* A similar situation was reflected in terms of the breakdown of some partnerships with other in-country NGOs. As one INGO’s competences and character evolved: “*[o]ur approach, and our partners’ approach just started to not mirror one another and how they wanted to work and how they felt the funds would be best spent wasn’t really aligned with* [our organisation’s] *approach”* [P13 SM].

The realignment of core activities within transforming NGOs meant there was a need to recruit new staff with the competence to fit the organisation for the requirements of the new institutional donor arena, as well as more experience of community development and participatory approaches. And, indeed, several participants in this study came into the equid sector from international development or the humanitarian sector. Changes also occurred at the top of organisations with some participants reporting rapid turnover of leadership creating instability in the organisations’ direction and the approaches that staff throughout the organisation were expected to put in place. As one field-based interviewee reported: *“[u]nfortunately the people coming in and bringing in all these ideas kept changing”* [P4 FS]. This was a disrupting period of confusion for staff working at ground level, especially those overseas working for INGOs who were not always fully informed about philosophy changes happening in head office: *“[s]o the poor old guys there, trying to work in a second language…with all these conflicting emails coming through saying why are you doing it that way, that’s not the way we do it. But last week that was the way we did it, now you want what?”* [P4 FS]. Another concern raised was that of being asked to repeat work each time the leadership changed: *“[s]coping is done every time a CEO comes…welfare assessments, basic issues, problems, prioritising the same thing. This is so wasteful of resources, and sometimes causes loss of experienced local staff”* [P11 FS]. From the point of view of addressing welfare needs, this was felt to be disruptive to welfare programming and hindered the progress of the initiatives already in place.

### Changes to organisational structure and programming autonomy

Organisational restructures also impacted other areas of programmatic planning. INGOs varied in the models of working that they utilised in overseas areas. Some had their own teams who were employed centrally by the organisation but consisted of individuals local to the target country. Others did not have their own overseas teams, instead opting to work with separate organisations that already existed in the target country. Some utilised a blend of both. This variety of models and organisational cultures was also reflected in Rogers *et al.* (2023) who described some of the associated challenges of cross-cultural working and the need for cultural sensitivity and knowledge of cultural variations in the perception of animal welfare. One challenge raised in the present study was the variation in the levels of autonomy that individuals working at ground level felt they had over the direction of their projects and the approaches implemented.

*“So sometimes there is some culture of…a shallow working with people on the ground. And so sometimes they say they have local contacts … sometimes that is very genuine and the partner organisation have a true voice and have a true input, and other times it’s not so genuine and they are really just kind of doing whatever they are told and as soon as the head office go away again they revert back”* [P5MM].

Historically, decisions regarding the approaches used across countries were made by individuals in the UK.

*“To me what happened then was we suddenly started trying to tell all the overseas teams exactly how to run their teams. Instead of letting them run their own team we started telling them what to do but at the same time we were telling them they had to run their own team and then we were saying ‘why have you done that?’ ‘Well because you told us to do what we thought was right’, ‘yeah but we didn’t say you could do that’”* [P4 FS].

In the current climate, participants generally described a shift away from this style of working. It was reported that there had been some positive reconfiguration of power dynamics between UK-based NGOs and local partners: *“I think probably the biggest change is a shift from a UK-centric way of working that implied we create the plans, we devise all of the strategies here in the UK then we send them to you in-country and you implement them.”* [P12 O]. However, with a move away from service provision approaches, some existing in-country programmes were terminated. The transition period was conducted differently by different organisations, with some phasing programmes out and others stopping services abruptly. A sudden ceasing of initiatives was perceived as putting field staff in a difficult position, being detrimental to animal welfare and being reputationally damaging for the organisation. This was powerfully articulated by P11 (FS):

*“[s]o many people actually calling me…I’ve seen this donkey having a car accident and leg broken, why don’t you come in actually take it and treat or if not recoverable euthanase it humanely? What do I answer to that organisation or that person if I have been told not to. This is something which is very, very difficult, and so damaging for the charity organisations.”*

In instances where decisions were made centrally in a top-down fashion, it was felt that the type of staff making programming decisions were likely to influence the practical feasibility of the suggested approaches. These factors could be related to the background of individuals: *“[t]he problem is always when an animal charity organisation is not run by animal welfare personnel, there is always a gap”* [P11 FS]. Factors relating to job role and level of field experience were also suggested: *“I guess you also have people that haven’t got the experience of international work, then you could find it quite easy to say ‘why don’t you just do this, surely that is easy to do’ and not realise actually how hard that is to implement in real terms”* [P2 FS].

A particularly productive form of partnership described was where an INGO partnered with a local NGO, that it could still be aligned to philosophically, that had autonomy over its programming and could be responsive to the ground needs: “*[t]hey* [INGO] *do not control anything, I mean,* [our organisation] *decide everything because we are working on the ground level”* [P25 MM]; similarly P17 (SM) suggested *“[i]t’s really driven by our project teams because…they’ve for the fountain of knowledge of their country, they know what the situation is like”.*

### Limitations and future opportunities

It should be noted that staff members and partner organisations included in this study are those that have remained after transitioning and restructuring processes and so may be more prepared to accept the disruption to the core activities of the organisation, limiting the range of perspectives documented. Another limitation of the study included fewer interviewees from small national NGOs in comparison to INGOs which may have led to a bias towards discussion of the types of challenges typically facing larger multi-national organisations, although data saturation was reached. Further work in this area could include animal welfare professionals across a wider range of countries, spoken languages and job roles to help determine whether similar views are shared on the topics discussed. As the findings are applicable to NGO programming more generally, it also presents an opportunity to take the approach beyond the field of animal welfare into other NGO sectors.

### Animal welfare implications

This study focuses on the perspectives of those working within animal welfare NGOs at multiple levels and their experiences regarding a spectrum of welfare programming approaches. When making decisions about what types of programmes to employ, NGOs want to ensure that animal welfare programming is maximally effective in terms of meeting animal needs. However, this involves key questions, such as trade-offs between the numbers of animals reached versus initiative sustainability. These are questions that are vital to the development of future welfare programming, not just for working equid welfare organisations (used as a lens for this study), but for the wider animal welfare field. The study provides insights into some of the challenges and influences that NGO staff face and discusses the implications of these for future programming development which will have direct impacts on animal welfare.

## Conclusion

Interviewing individuals from a variety of roles across organisations allowed a wide range of perspectives to be included and gave staff a space to voice their opinions.

Organisations felt the need to be responsive and adapt to the changing external landscape and policy environment. However, maintaining a balance between direction implementation and advocacy-based activities came through strongly as a theme, with staff at either end of this programming spectrum believing that their role was helping to achieve their charity’s mission and ethos. Both changing and not changing programming direction came with associated risks and benefits.

Risks of changing included the creation of tension where the values of individuals who supported direct implementation approaches did not align with the new programming direction; this perceived mission drift led in some instances to the loss of staff members, organisational partners and individual donors. Partnering with larger organisations from other sectors or pursuing institutional funding increased upwards accountability requirements. Difficulties were encountered, when working internationally, balancing decentralisation and the devolution of control over programming direction. Difficulties for staff and previous beneficiaries were experienced in transition periods when phasing out direct implementation. The potential was also described for limited impact due to external factors affecting the policy environment or a lack of infrastructure resulting in poor policy enforcement. Risks of not changing included poor long-term sustainability, treating the symptoms rather than the root causes of welfare problems, high costs, a lack of community ownership and engagement with initiatives, the creation of dependency on NGO services and the undermining of local infrastructure.

We argue that the sustainability of equid programming (in terms of reach and effectiveness) is best achieved via a hybrid organisational structure. Retaining some direct implementation, which may be in partnership with locally based autonomous NGOs (allowing field staff the ability to tailor initiatives to local conditions), alongside high-level advocacy work appears to be the optimum position on the direct implementation-advocacy spectrum. We argue that a wholesale move to advocacy-only programming carries risks in contexts where external factors may derail programming efforts (for example, government instability). Whilst having to respond dynamically to their environment, NGOs need to evaluate the risks associated with programming decisions, considering their vision, mission and staff identity. Using institutional analysis can provide a valuable insight into the transformations that NGOs are undergoing, from a variety of different perspectives.

## Supporting information

Haddy et al. supplementary material Haddy et al. supplementary material

## Supplementary material

The supplementary material for this article can be found at http://doi.org/10.1017/awf.2024.11.
