# Supplementary material for: “We forgot about the donkeys!” An institutional analysis of the shift in animal welfare from direct implementation towards advocacy-based programming
Source: Anim Welf. 2024 Feb 14;33:e9. doi: 10.1017/awf.2024.11 (PMC10951660; doi:10.1017/awf.2024.11)
Supplement: Haddy et al. supplementary material [file S0962728624000113sup001.pdf]

Semi Structured Interview Themes

1) **Brief Work Profile**
- Can you briefly describe your current role and how long you have worked within the organisation?

2) **Previous Experience of equid welfare initiatives**
- Have you utilised any of the different types of welfare intervention approaches on the pre-interview list?

- Can you talk me through the approaches you have experience of using and under what circumstances you have used them?

- Which have been the most (and least) successful?

- In your opinion what are the potential reasons for/conditions that have resulted in this success or lack of it? (These could potentially be structural within the organisation, environmental, determined by the most prevalent types of welfare problems, financial, due to the social dynamics of the target community, or other factors)

- What is your opinion regarding the potential utility of participatory approaches including Participatory Rural Appraisal (PRA) exercises as an alternative to more traditional educational approaches? Do you think PRA exercises have any particular advantages or disadvantages and do you think they would be useful in your area?

- Do you think that variations in culture and owner attitudes could result in interventions that are successful in one part of the world being unsuccessful in another?

3) **Approaches within the participant's current organisation**

- What are the most commonly used approaches within the organisation?

- Do you think that the welfare approaches chosen by the organisation's managers are always able to be practically implemented?

- Within the time you have worked for the organisation, have you seen changes in the types of initiatives engaged in by the organisation and in your opinion are these changes for the better or not?

- Why do you think these changes have been made?

- Do you think that the approaches currently used in the organisation would be those that would be employed if you were given an unlimited budget to run welfare initiatives?

- If you were in charge of the organisation, what initiatives would you focus your resources on and why?
